# Atypical presentation of glomus tumor in the facial superficial vein on high-frequency ultrasound: a case report

**DOI:** 10.3389/fmed.2026.1790426

**Published:** 2026-05-20

**Authors:** Ting Zhang, Jiaojiao Zhou

**Affiliations:** Department of Medical Ultrasound, West China Hospital, Sichuan University, Chengdu, China

**Keywords:** case report, facial neoplasm, glomus tumor, high-frequency ultrasound, superficial vein

## Abstract

Glomus tumors are rare mesenchymal tumors that typically occur in the extremities, particularly the intravascular subtype. We present a case report of a 28-year-old male with a painless firm nodule on the left cheek. High-frequency ultrasound (HFUS) revealed a mass within a superficial vein of the left cheek, characterized by well-defined margins, a regular shape, heterogeneous echogenicity, and relatively rich internal vascularity. These ultrasound features were highly similar to the typical imaging of intravascular papillary endothelial hyperplasia, leading to initial misdiagnosis. Following clinical evaluation and per the patient’s request for definitive treatment, surgical resection was performed. Histopathological and immunohistochemical analysis confirmed the diagnosis of a glomus tumor originating from a facial superficial vein. In conclusion, this rare superficial venous glomus tumor presented with atypical clinical and imaging features, notably the absence of typical pain and distinct differences from the imaging characteristics of extremity glomus tumors. This case report aims to improve the recognition of such lesions and prevent misdiagnosis between intravascular papillary endothelial hyperplasia and facial superficial venous glomus tumors.

## Introduction

Glomus tumors are classified as vascular neoplasms of perivascular origin, accounting for approximately 2% of all soft tissue tumors (1). The majority of these tumors are benign, with malignant variants being exceptionally rare (2). They typically present as solitary lesions; however, approximately 10% manifest as multiple lesions, most frequently located in the nail beds, cutaneous tissues, or soft tissues of the extremities. Rare occurrences have been documented in visceral organs or superficial veins (3, 4). These tumors are typically characterized by small nodules associated with localized tenderness, cold sensitivity, and intense, paroxysmal pain. Although most glomus tumors are small (with a maximum diameter of less than 4 cm), tumors in rare cases involving visceral organs can grow much larger (5, 6).

In terms of diagnosis, high-frequency ultrasound (HFUS) enables real-time, high-resolution detection of small glomus tumors without requiring intravenous contrast administration, while providing detailed visualization of lesional blood flow and surrounding tissue architecture (7). Nevertheless, definitive diagnosis mainly relies on histopathological examination supplemented by immunohistochemistry (IHC) (8). We herein report a case of superficial venous glomus tumor, which lacked the typical clinical manifestations of glomus tumors, demonstrated HFUS features highly suggestive of intravascular papillary endothelial hyperplasia (IPEH), and was finally confirmed as a glomus tumor by IHC. We present this case to supplement the data of atypical HFUS imaging manifestations of such lesions, emphasize the need to combine imaging features with IHC results to ensure diagnostic accuracy, and enrich the clinical data of rare lesions such as superficial venous glomus tumors, to provide a reference for clinical differential diagnosis.

## Case presentation

**FIGURE 1:**
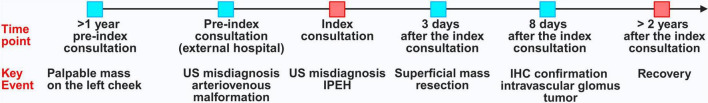
Timeline of clinical diagnosis and treatment.

A 28-year-old man presented with a palpable mass on the left cheek, which he first noticed more than 1 year prior to consultation and had not received special treatment due to the absence of pain, cold sensitivity or other discomfort (Figure 1). His medical history was unremarkable, with no prior trauma to the affected area, no history of cutaneous neoplasms, and no chronic systemic diseases associated with soft tissue lesions. Additionally, there was no family history of similar skin or soft tissue masses, or ideritary conditions predisposing to such lesions. Physical examination revealed a mobile, non-tender nodule without cold sensitivity. A slight brownish pigmentation, measuring 0.2 cm in diameter, was observed on the overlying skin, and no vascular bruit was detected on auscultation. An initial ultrasonographic evaluation performed at a referring hospital was suggestive of an arteriovenous malformation.

**FIGURE 2:**
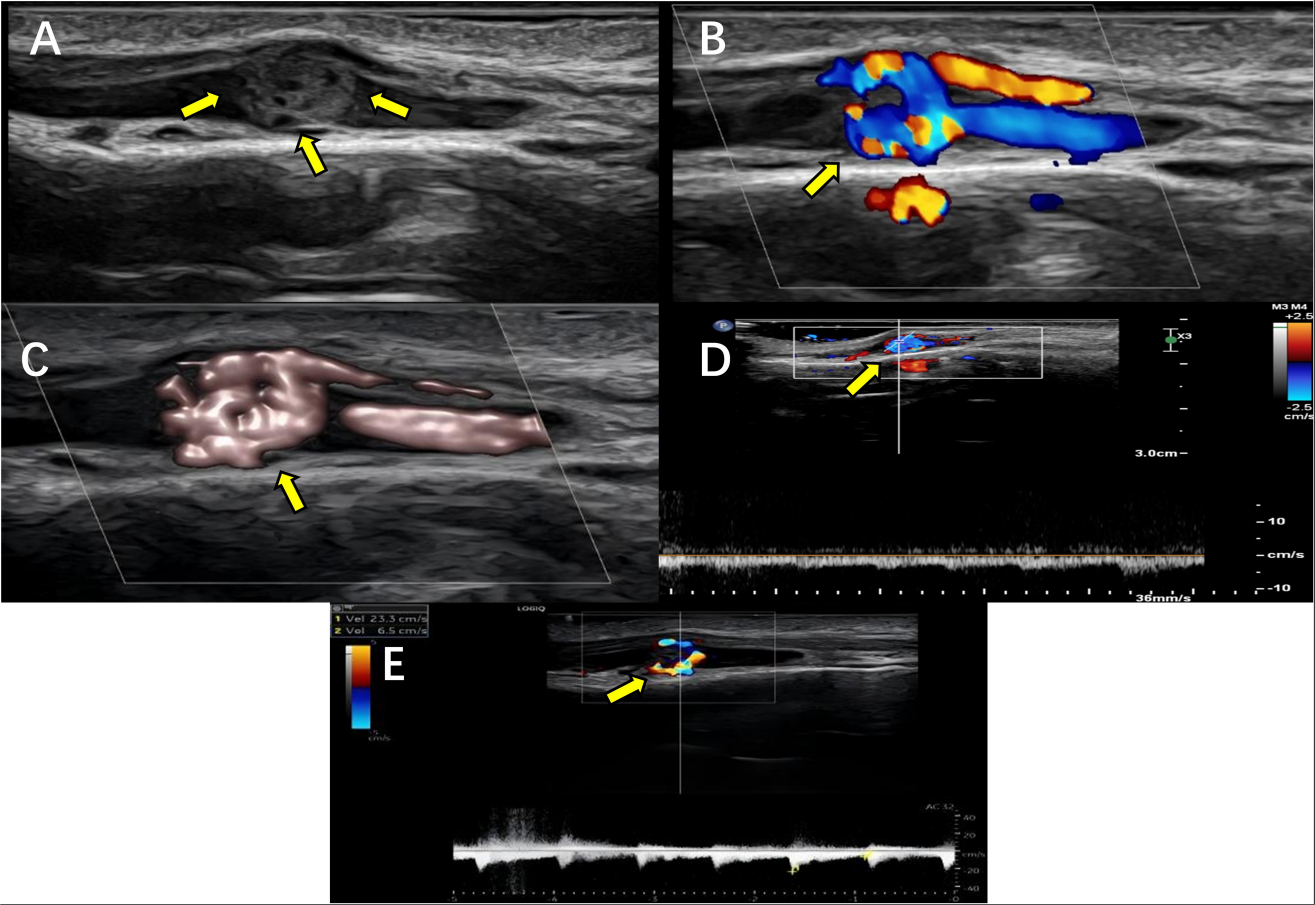
Multimodal ultrasonic manifestations of benign glomus tumor in subcutaneous superficial vein. **(A)** Grayscale ultrasound. **(B)** Color Doppler imaging. **(C)** Power Doppler imaging. **(D)** Venous spectrum was detected within the mass. **(E)** Arterial spectrum was detected in the mass.

Two-dimensional gray-scale ultrasonography reveals a solid intraluminal nodule within the venous lumen (Figure 2A). The nodule presents a round or oval shape, well-circumscribed borders, regular morphology, and tubuloreticular architecture of internal echoes. The affected vein shows mild dilatation, and the nodule has excellent continuity with the venous wall without evidence of obvious extraluminal invasion. Upon compression with the ultrasound transducer, the venous lumen can be completely compressed and collapsed, whereas the tumor itself exhibits poor compressibility. On Color Doppler Flow Imaging (CDFI), abundant intralesional vascularity is detected within the nodule, presenting as an interlaced red-and-blue reticular blood flow pattern (Figures 2B, C). Pulsed-wave (PW) Doppler identifies both venous-type spectra and low-velocity, high-resistance arterial flow spectra. Adequate blood flow filling is visualized in the dilated superficial veins at the proximal and distal ends of the tumor (Figures 2D, E).

According to HFUS, a diagnosis of IPEH was initially considered. Given the small size of the patient’s facial lesion and its imaging features on HFUS, a multidisciplinary consultation involving radiologists, sonographers, and plastic surgeons determined that additional imaging (e.g., MRI or CT) was not indicated. Additionally, considering the patient’s active request for radical treatment, it was ultimately decided to perform surgical resection on the patient.

**FIGURE 3:**
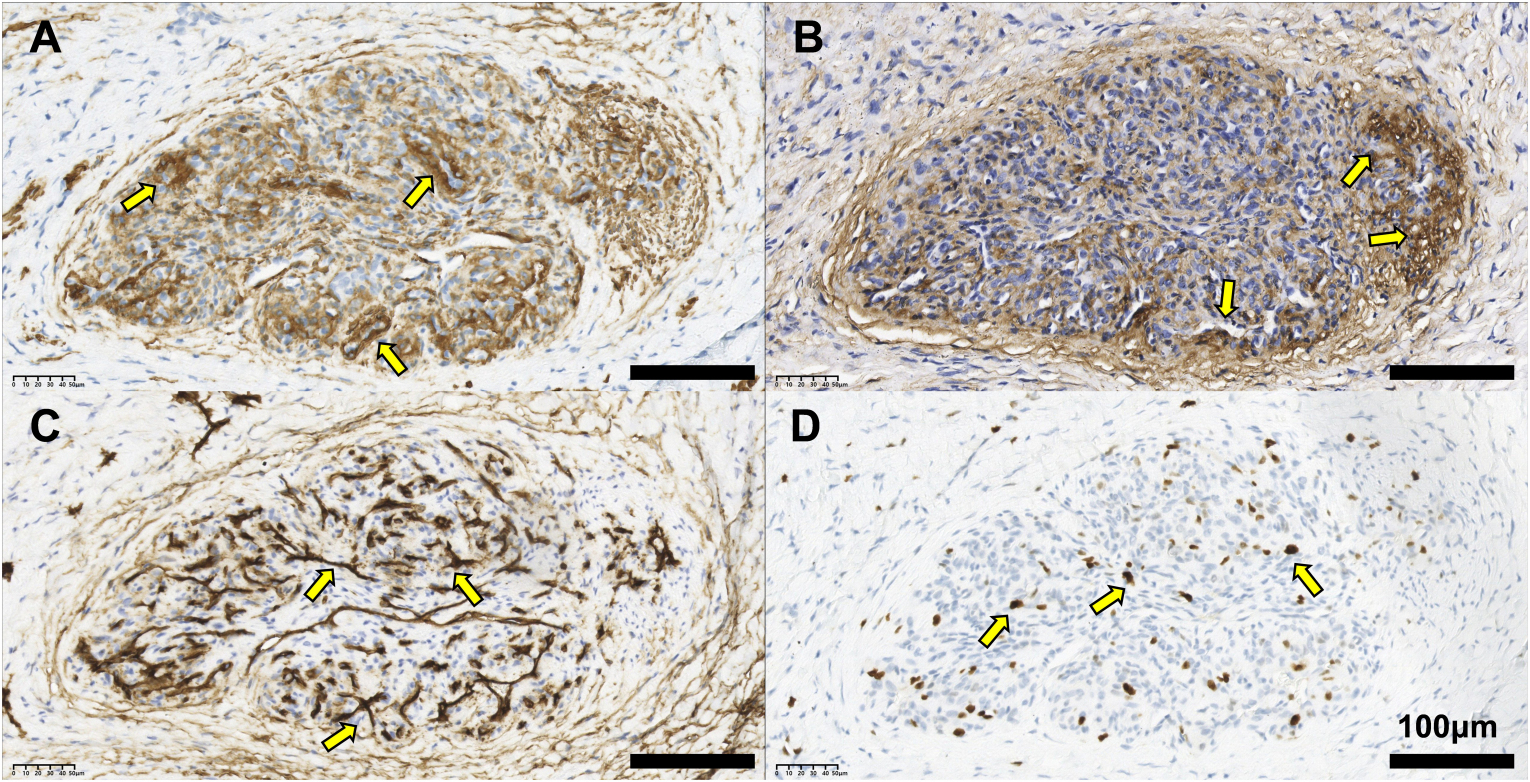
The pathological manifestations of glomus tumor in superficial vein. **(A)** Immunohistochemistry (IHC) showing the expression of SMA (30 × magnification).**(B)** IHC showing the expression of Collagen IV (30 × magnification).**(C)** IHC result showing theexpression of CD34 (30 × magnification). **(D)** IHC result showing the expression of Ki-67(MIB-1) (+, approximately 10%) (30 × magnification).

Superficial mass resection combined with fascial flap plasty was performed under local anesthesia via a linear skin incision along skin tension lines. The lesion was completely resected en bloc, with histopathologically confirmed negative circumferential surgical margins. Following resection, gross pathological examination of the surgical specimen revealed an irregular, fusiform tissue mass measuring 0.6 × 0.4 × 0.4 cm. A non-protruding gray-brown area (0.2 cm in diameter) was identified in the central region of the specimen; the cut surface was solid, gray-white to gray-brown, and of moderate consistency. IHC showed SMA (+) (Figure 3A), Collagen IV (+) (Figure 3B), CD34 (vessels+) (Figure 3C), Ki-67 (MIB-1) approximately 10% (Figure 3D), and Caldesmon (−), Desmin (−), S100 (−), Synaptophysin (−), CK5(−), CK6 (−), and CK7 (−). These findings supported the diagnosis of the glomus tumor. These immunophenotypic findings were fully consistent with the diagnosis of benign intravascular glomus tumor.

The patient had an uneventful postoperative recovery, with good patient-reported recovery experience, and no new lesions or signs of recurrence during more than 2 years of regular follow-up.

## Discussion

Glomus tumors originate from glomus cells, specialized smooth muscle cells, that surround the anastomotic vessels of the glomus body. It predominantly distributed in the subungual regions of fingers and toes, palms, and soles, where it participates in thermoregulation. Aberrant proliferation of these cells, triggered by genetic predisposition, trauma, or inflammation, can lead to tumor formation (8). Glomus tumors are stratified according to their malignant potential into benign, malignant, or uncertain categories, and also by numerical occurrence. The multiple variant is notably often hereditary and has a higher prevalence in the pediatric population (9, 10). Although most occur in extremities, they have also been reported in visceral organs such as the stomach (11, 12), knee (13), trachea, and breast (14). The superficial facial glomus tumor in our case had a maximum diameter of only 0.6 cm, which was notably smaller than the typically larger visceral variants reported in previous studies. This finding suggests that there may be a certain correlation between tumor size and anatomical location. However, tumors confined to veins are uncommon, and they can disseminate intravascularly, sometimes forming multiple extensions (15).

The classic symptom triad-pain, pinpoint tenderness, and cold hypersensitivity-is reported in 63%–100% of subungual cases (16). Superficial lesions often present as bluish, palpable nodules. Imaging modalities for glomus tumors include MRI, X-ray, CT, and ultrasound (17). For superficial or subungual lesions, ultrasound is faster, more convenient, and real-time compared to other modalities (18). Of the three primary imaging modalities for glomus tumors, HFUS serves as the first-line, non-invasive method for visualizing smaller and superficial tumors. Meanwhile, MRI provides superior soft-tissue resolution, typically revealing T2 high-signal intensity, which is ideal for atypical or deep lesions; while X-ray may only show indirect signs like bone erosion and cannot directly visualize the tumor itself.

Based on the imaging features of HFUS, systematic differential diagnosis can be performed between intravascular glomus tumor and common vascular lesions of the superficial soft tissues (Table 1). The key differential diagnoses include venous malformation (VM), arteriovenous malformation (AVM), angioleiomyoma (19), and IPEH (20, 21). VM and AVM can be clearly differentiated from the index lesion via ultrasound. VM typically manifests as tortuous, dilated, compressible anechoic venous lumens in the subcutaneous tissue, with only venous flow spectra detectable and no evidence of a solid space-occupying lesion. AVM presents as ill-defined, tortuous tubular or honeycomb-like hypoechoic or anechoic areas in the subcutaneous tissue, filled with a chaotic mosaic pattern of color Doppler flow signals; enlarged feeding arteries can be observed entering the vascular network, along with tortuous dilated draining veins exiting the network, within which low-resistance arterial spectral waveforms are detected. Both entities have markedly distinct sonographic features from the index lesion, which is characterized by a well-circumscribed solid mass within the venous lumen. The index lesion is readily differentiated from angioleiomyoma: the latter is typically located perivascularly or within the vessel wall rather than inside the vascular lumen, and sonographically appears as a solid perivascular nodule without intraluminal mass effect within the vein. IPEH represents the primary differential diagnostic challenge for this lesion, with substantial overlap in sonographic features between the two entities. Both may present as hypoechoic or mixed-echoic solid nodules within the venous lumen, frequently accompanied by abundant internal vascularity. Reliable differentiation cannot be achieved by high-frequency ultrasound alone, and definitive diagnosis ultimately relies on histopathological and immunohistochemical examination.

**TABLE 1:** Systematic differential diagnosis.

Condition	Ultrasound findings
VM	It occurred in the skin, subcutaneous tissue, and muscles. It appeared as an ill-defined hypoechoic or anechoic lesion without a distinct capsule. Slow venous flow was seen within the anechoic area. Compression causes lumen collapse and increased flow signals. In thrombosis, flow signals were absent, and hyperechoic foci (phleboliths) may be seen.
AVM	The subcutaneous tissue was thickened and hyperechoic, with irregular hypoechoic/anechoic areas showing a reticular or honeycomb pattern. These areas demonstrated mosaic flow signals and high-velocity, low-resistance arterial spectra. Findings included dilated feeding arteries, and tortuous veins with arterial-like flow.
Angioleiomyoma	A well-defined, relatively regular, round or oval hypoechoic mass in the skin or subcutaneous tissue. The internal echogenicity may be homogeneous or heterogeneous. Abundant blood flow signals can be detected within most of these masses (19).
IPEH	Ultrasound shown an irregular, well-defined hypoechoic intravascular mass with heterogeneous echotexture. It typically exhibits marked internal vascularity (dendritic or spot-like/linear flow). The involved vessel failed to collapse completely with probe compression (20, 21).

This case of intravascular glomus tumor was exceptionally rare, and its atypical clinical presentation posed extremely diagnostic challenges. The patient presented solely with a painless soft tissue mass and other imaging modalities were deemed unsuitable. HFUS clearly demonstrated the lesion within the venous lumen, showing a homogeneous hypoechoic pattern with relatively abundant internal blood flow and a detectable arteriovenous spectrum. These sonographic features highly suggested a vascular lesion. Combined with the absence of pain, that initially led to the misdiagnosis of intravascular papillary endothelial hyperplasia. IPEH, also known as Masson’s tumor, is a benign intravascular lesion, pathologically characterized by the organization of thrombus accompanied by a peculiar form of benign papillary endothelial hyperplasia (22). Sonographically, it also frequently presents as an oval or lobulated hypoechoic or mixed-echoic nodule or mass, which can likewise exhibit abundant internal blood flow (21). These two entities demonstrated significant overlapping features on ultrasound imaging, making their differential diagnosis particularly difficult when they occurred within vein.

Surgical excision is the only curative treatment for glomus tumors, and en bloc resection with negative surgical margins is the standard of care to prevent recurrence. For our patient, complete surgical resection was performed after shared decision-making with the patient. Postoperative histopathological and immunohistochemical examination confirmed the final diagnosis of benign intravascular glomus tumor, ruled out rare malignant potential, and provided a definitive etiological diagnosis. The patient has remained recurrence-free throughout the follow-up period, consistent with the excellent prognosis of benign glomus tumors after complete resection.

### Limitations and clinical lesson

This is a single-case report, and the generalizability of the study findings is inherently limited. In addition, the present study lacks correlative analysis with additional imaging modalities.

This case offers critical, practice-guiding clinical insights for the diagnosis of rare superficial soft tissue tumors. Most importantly, our findings confirm that HFUS alone cannot reliably differentiate intravascular glomus tumor from IPEH, given the extensive overlap in their sonographic features. Histopathological examination therefore remains the irreplaceable cornerstone for definitive diagnosis of these lesions. This core takeaway reminds clinicians to prioritize standardized histopathological and immunohistochemical evaluation for superficial masses with atypical imaging features, to minimize the risk of misdiagnosis in routine clinical practice.

## Conclusion

We report a rare case of superficial intravascular facial glomus tumor with atypical clinical and imaging features. The lesion lacked the classic pain triad of glomus tumors, showed significant sonographic overlap with IPEH leading to initial misdiagnosis, and was ultimately confirmed by histopathological and immunohistochemical examination. This report supplements the clinical and imaging data of atypical rare glomus tumors, clarifies the core differential diagnostic pitfalls between intravascular glomus tumor and IPEH, and highlights the essential role of histopathology in definitive diagnosis, with the ultimate goal of reducing misdiagnosis and improving diagnostic accuracy for similar rare lesions.

## Data Availability

The original contributions presented in this study are included in this article/supplementary material, further inquiries can be directed to the corresponding author.
